# Burnout among Healthcare Workers in Saudi Arabia After COVID-19: A Systematic Review

**DOI:** 10.1192/j.eurpsy.2026.11830

**Published:** 2026-07-31

**Authors:** M. T. Alotaibi, M. S. Almutairi, S. S. Alotaibi, A. M. Bukhary, M. I. Almatroudi, A. A. Alfattani, S. S. Enani, A. M. Aljarad, A. A. Almarshedi

**Affiliations:** 1 Psychiatry; 2 Psychology, PSMMC; 3 Biostatistics, Epidemiology and Scientific Computing, KFSHRC, Riyadh, Saudi Arabia

## Abstract

**Introduction:**

A resilient clinical workforce is crucial to ensuring high-quality care and equitable access. Since COVID-19, concerns about burnout among healthcare workers (HCWs) have grown.

**Objectives:**

This systematic review aims to (1) estimate the prevalence of overall burnout among HCWs in Saudi Arabia after March 2020; and (2) appraise how burnout is measured.

**Methods:**

A systematic search of MEDLINE/PubMed, Embase, PsycINFO, Web of Science, Google Scholar, and the Saudi Digital Library was conducted to identify studies of healthcare workers in Saudi Arabia that collected data after March 2020 assessing burnout using validated tools. Eligible designs were cross-sectional or longitudinal studies reporting overall prevalence or subscale estimates (emotional exhaustion, depersonalization, personal accomplishment). Risk of bias was assessed with a modified Newcastle-Ottawa Scale. Although meta-analytic pooling was planned, heterogeneity in ascertainment methods and definitions rendered pooled estimates unreliable, and studies were summarized descriptively. A prespecified subset of larger studies (n >200, full-length instrument, explicit cutoffs) was analyzed separately.

**Results:**

Burnout prevalence data were extracted from 56 studies involving 21,220 individuals in 7 region across Saudi Arabia, published between 2021 and 2025. In total, 67.9% (38/56) of studies employed a version of the Maslach Burnout Inventory (MBI) to assess burnout, 28.6% (16/56) used the Copenhagen Burnout Inventory (CBI), and 3.6% (2/56) utilized other instruments. Studies have reported varying prevalence estimates of overall burnout or its subcomponents: 42.9% (24/56) for overall burnout, 60.7% (34/56) for emotional exhaustion, 60.7% (34/56) for depersonalization, and 60.7% (34/56) for low personal accomplishment. Studies used at least 50 unique definitions for meeting overall burnout or burnout subscale criteria. Studies variably defined burnout based on predefined cutoff scores and used markedly different cutoff definitions. Among studies using instruments based on the MBI, there were at least 13 distinct definitions of overall burnout prevalence and 9, 8, and 9 definitions of emotional exhaustion, depersonalization, and low personal accomplishment prevalence, respectively. The overall prevalence of burnout ranged from 27.3% to 89.1%. The prevalence of emotional exhaustion, depersonalization, and low personal accomplishment. ranged from 12.6% to 78.1%, 10.6% to 98.0%, and 8.4% to 100.0%, respectively.

**Conclusions:**

The prevalence of burnout among HCWs in Saudi Arabia varied widely, and there was marked variation in burnout definitions, measurement approaches and study quality which precluded definitive conclusions about the prevelance of burnout. Future research should utilize a consensus definition and standardized measurement using validated instruments with explicit cutoffs to enable national monitoring and guide targeted workforce support.

**Disclosure of Interest:**

None Declared

